# Methylene-Bridged Tridentate Salicylaldiminato Binuclear Titanium Complexes as Copolymerization Catalysts for the Preparation of LLDPE through [Fe]/[Ti] Tandem Catalysis

**DOI:** 10.3390/polym11071114

**Published:** 2019-07-01

**Authors:** Yani Luo, Jian Li, Derong Luo, Qingliang You, Zifeng Yang, Tingcheng Li, Xiandan Li, Guangyong Xie

**Affiliations:** 1 Hubei Key Laboratory of Catalysis and Materials Science, School of Chemistry and Materials Science, South-Central University for Nationalities, Wuhan 430074, China; 2 Key Laboratory of Optoelectronic Chemical Materials and Devices, Ministry of Education, School of Chemical and Environmental Engineering, Jianghan University, Wuhan 430056, China

**Keywords:** tandem catalysis, in-situ copolymerization, linear low-density polyethylene, non-metallocene, binuclear titanium complex

## Abstract

A novel tandem catalysis system consisted of salicylaldiminato binuclear/mononuclear titanium and 2,6-bis(imino)pyridyl iron complexes was developed to catalyze ethylene in-situ copolymerization. Linear low-density polyethylene (LLDPE) with varying molecular weight and branching degree was successfully prepared with ethylene as the sole monomer feed. The polymerization conditions, including the reaction temperature, the Fi/Ti molar ratio, and the structures of bi- or mononuclear Ti complexes were found to greatly influence the catalytic performances and the properties of obtained polymers. The polymers were characterized by differential scanning calorimetry (DSC), high temperature gel permeation chromatography (GPC) and high temperature ^13^C NMR spectroscopy, and found to contain ethyl, butyl, as well as some longer branches. The binuclear titanium complexes demonstrated excellent catalytic activity (up to 8.95 × 10^6^ g/molTi·h·atm) and showed a strong positive comonomer effect when combined with the bisiminopyridyl Fe complex. The branching degree can be tuned from 2.53 to 22.89/1000C by changing the reaction conditions or using different copolymerization pre-catalysts. The melting points, crystallinity and molecular weights of the products can also be modified accordingly. The binuclear complex **Ti^2^L_1_** with methylthio sidearm showed higher capability for comonomer incorporation and produced polymers with higher branching degree and much higher molecular weight compared with the mononuclear analogue.

## 1. Introduction

**Figure 1:**
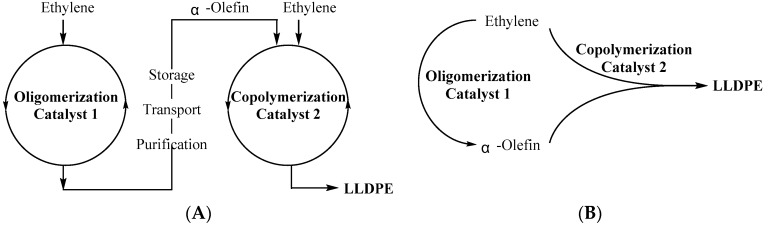
The diagrammatic sketch of two different methods to product LLDPE. (**A**) two-step method; (**B**) tandem catalysis.

Polyethylene contributes to about 50% of the global polyolefin consumption, and one of the fastest growing types of polyethylene is linear low density polyethylene (LLDPE), which is widely applied in film production for the packaging industry because of its high tear and impact strength. LLDPEs are generally made by a two-step method from the copolymerization of ethylene and α-olefin comonomers such as 1-butene, 1-hexene and 1-octene catalyzed by conventional heterogeneous Ziegler-Natta or metallocene catalysts, where the comonomer α-olefins were commonly generated from ethylene oligomerization (Figure 1A). An alternative approach to the synthesis of LLDPE is the use of tandem catalysis, where one catalyst oligomerizes ethylene to α-olefin and the other simultaneously copolymerizes excessive ethylene with the in situ produced α-olefin (Figure 1B). Such single-step process has obvious advantages over traditionally used two-stage process in terms of the costs of plant investment, α-olefin purification, storage, and transport.

Tandem catalysis is also called in-situ copolymerization and involves the cooperative action of oligomerization and copolymerization catalyst precursors in one reactor. Since it was first reported by Beach and Kissin in the 1980s [1,2], a wide variety of promising tandem catalytic systems involving two different Ziegler-Natta catalysts [1,2,3,4], Ziegler-Natta catalyst combined with metallocene [5] or nonmetallocene catalyst [6,7], and one [8,9] or two [10,11,12] metallocene catalysts have been developed to prepare branched polyethylene in situ. Moreover, numerous investigations were carried out on tandem catalytic systems comprising metallocene and non-metallocene catalysts [13,14,15,16,17,18,19,20,21,22,23,24,25,26,27,28,29]. Nevertheless, the copolymerization catalysts in these tandem systems were almost Ziegler-Natta or metallocene catalysts.

**Scheme 1:**
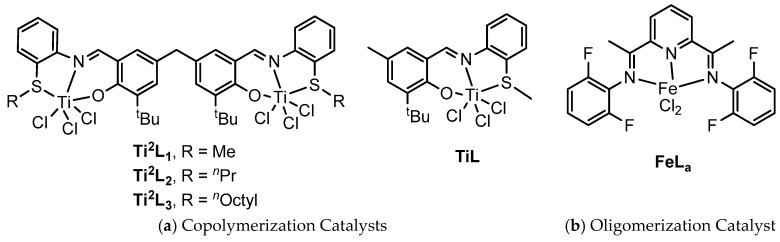
Tandem catalytic system.

In the last twenty years, group 4 non-metallocene catalysts have attracted a great deal of attention due to their excellent performances not only for ethylene polymerization but also for the copolymerization of ethylene with other olefins [30,31,32,33,34,35]. Lately, binuclear and multinuclear complexes have also been researched for olefin polymerization, which showed that the cooperative action of two or more nearby metal centers could significantly increase catalytic activity, modify molecular weight and branching degree, and enhance comonomer incorporation selectivity [36,37,38,39,40,41,42,43,44,45,46,47,48]. Among these, early transition non-metallocene binuclear complexes have rarely been reported. Our lab has been focused on developing novel non-metallocene catalysts through modification of the coordination environment of the central metals by using the steric/electronic effect of the substituents [49,50,51,52,53,54,55,56]. Recently we have successfully prepared LLDPE from ethylene in a single reactor using non-metallocene titanium complex as the copolymerization catalyst of a tandem dual-functional catalyst systems [57,58]. We have also designed and synthesized a number of non-metallocene binuclear titanium complexes which demonstrated excellent activity for ethylene (co)polymerization [59,60,61,62]. Here we used the non-metallocene binuclear titanium complexes for the first time as copolymerization catalysts of a tandem catalyst system and described the direct synthesis of LLDPE from ethylene in a single reactor though [Fe]/[Ti] tandem catalysis, which was shown in Scheme 1. Compared with other reported tandem catalysts, this [Fe]/[Ti] tandem system showed superior synergistic catalysis, strong positive comonomer effect, high catalytic activity, and good structural adjustability of copolymers. The copolymerization activities demonstrated by our binuclear Ti/bisiminopyridyl Fe system were among the highest reported.

## 2. Materials and Methods

### 2.1. Materials

All manipulation involving air- and/or moisture-sensitive compounds was carried out under purified nitrogen using standard vacuum-line. Toluene purified through distillation with sodium benzophenone ketyl under nitrogen. MMAO (7% aluminum in a heptane solution) was acquired from Akzo Nobel Chemical, Inc. (Amersfoort, The Netherlands). All other chemicals are used as received from commercial sources. Fe complex **FeL_a_** and binuclear Ti complexes **Ti^2^L_1_**, **Ti^2^L_2_** and **Ti^2^L_3_** were prepared according to our previous works [58,59].

The mononuclear Ti complex **TiL** was synthesized with a similar procedure as for the binuclear analogue. Yield 91%. ^1^H NMR (400 MHz, CDCl_3_): δ 8.74 (d, *J* = 32.9 Hz, 1H, CH=N), 7.66 (t, *J* = 8.0 Hz, 1H, Ar*H*), 7.59–7.35 (m, 5H, Ar*H*), 3.09 (d, *J* = 23.8 Hz, 3H, SC*H*_3_), 2.43 (d, *J* = 8.5 Hz, 3H, C*H*_3_), 1.53 (d, *J* = 3.6 Hz, 9H, C(C*H*_3_)_3_). ^13^C NMR (101 MHz, CDCl_3_): δ 163.32 (*C*H=N), 160.91, 150.44, 137.27, 136.62, 134.62, 134.28, 133.01, 131.13, 130.83, 130.43, 127.76, 119.10, 35.18 (*C*(CH_3_)_3_), 29.81 (C(*C*H_3_)_3_), 27.53 (*C*H_3_), 20.99 (S*C*H_3_). IR(KBr, cm^−1^): 3356, 2959, 1591, 1545, 1379, 1327, 1259, 863, 757, 634, 604. Anal. Calcd for C_19_H_22_Cl_3_NOSTi: C, 48.90; H, 4.75; N, 3.00%. Found: C, 49.21; H, 4.58; N, 3.32%.

### 2.2. Characterization

The ^1^H NMR spectra of ligands and complexes were measured on a Bruker Avance III 400 MHz spectrometer (Karlsruhe, Germany). Elemental analyses were performed with Vario EL 111 elemental analyzer. The ^13^C NMR data of the ethylene/α-olefin copolymers were obtained on a Varian XL 300 MHz spectrometer at 120 °C with *o*-C_6_D_4_Cl_2_ as the solvent. Differential scanning calorimetry (DSC) measurements were performed on a Netzsch DSC200 F3 instrument (Bavaria, Germany). Each sample was heated from 30 to 200 °C at a heating rate of 10 °C/min and reheated at the same rate. The *M*_n_ and *M*_w_/*M*_n_ of the polymers were determined at 150 °C with a Viscotek 350A HT-GPC System (San Jose, CA, USA) using a polystyrene calibration. 1,2,4-Trichlorobenzene was employed as the solvent at a flow rate of 1.0 mL/min.

### 2.3. Ethylene In-Situ Copolymerization

A flame-dried Schlenk flask purged with N_2_ was filled with ethylene gas. 40 mL of freshly distilled toluene was added and raised to the reaction temperature for 10 min. MMAO was then injected using a syringe and the mixture was stirred for 5min. Copolymerization was initiated by injection of the solutions of [Fe] and [Ti] catalysts at the same time. The polymerization was quenched with 100 mL acidified ethanol (10 vol.% HCl) after a desired time. The product was precipitated, filtered, then washed with ethanol, and dried at 50 °C to constant weight under vacuum. 

## 3. Results and Discussion

The methylene-bridged tridentate salicylaldiminato binuclear titanium complexes displayed extremely high activities for ethylene polymerization and ethylene/α-olefins copolymerization at atmospheric pressure in our previous research [59], thus may be excellent candidates as copolymerization catalysts of dual-function catalytic systems for ethylene in-situ copolymerization. Here we investigated the tandem catalytic behaviors of non-metallocene binuclear titanium complexes (**Ti^2^L_1_**-**Ti^2^L_3_**) and bisiminopyridyl iron complex (**FeL_a_**) with ethylene as the only feed, and the results were listed in Table 1.

The Ti complex **Ti^2^L_1_** and Fe complex **FeL_a_** exhibited high catalytic activity for ethylene polymerization or oligomerization when used separately (Entries 1 and 2 of Table 1), which has been detailedly investigated in our previous works [58,59], and the binuclear titanium complexes still displayed very high activity when combined with bisiminopyridyl Fe complex in this tandem catalytic system. The effects of the reaction temperature, [Fe]/[Ti] molar ratios and catalyst structures upon the copolymerization activities and properties of the copolymers were investigated. The melting temperatures, branching degrees and molecular weights were measured by DSC, ^13^C NMR and GPC, respectively. 

**Figure 2:**
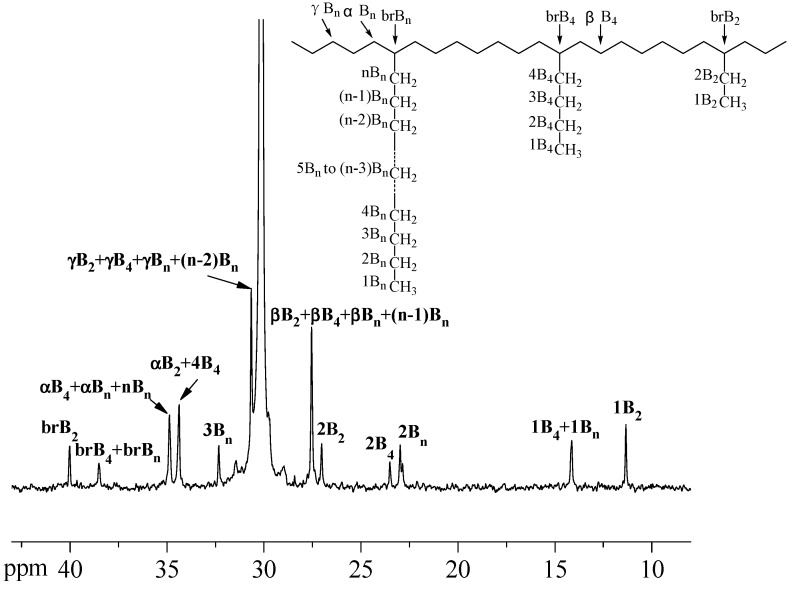
Typical ^13^C NMR spectrum of the LLDPE by **1**/**2**/MMAO tandem system (Run 6 in Table 1).

Compared with the catalysis by Ti/MMAO, the tandem catalysis system (Fe/Ti/MMAO) produced polyethylene with much reduced melting temperature (*T*_m_) and crystallinity (*X*_c_), which implied that the polymer contained considerable amount of branched chain that must come from the in-situ copolymerization of ethylene with α-olefins produced by Fe complex. The branching structures were further confirmed by high temperature ^13^C NMR spectrum as shown in Figure 2. Various types of branches, including ethyl branches (δ 11.19, 26.87, 39.86 ppm), butyl branches (δ 23.36, 29.57 ppm) and some longer branches with six or more carbons (δ 13.98, 22.83, 32.17, 38.35 ppm) were found, and the branching degrees were calculated according to ref. [63].

### 3.1. Influence of Reaction Temperature

**Figure 3:**
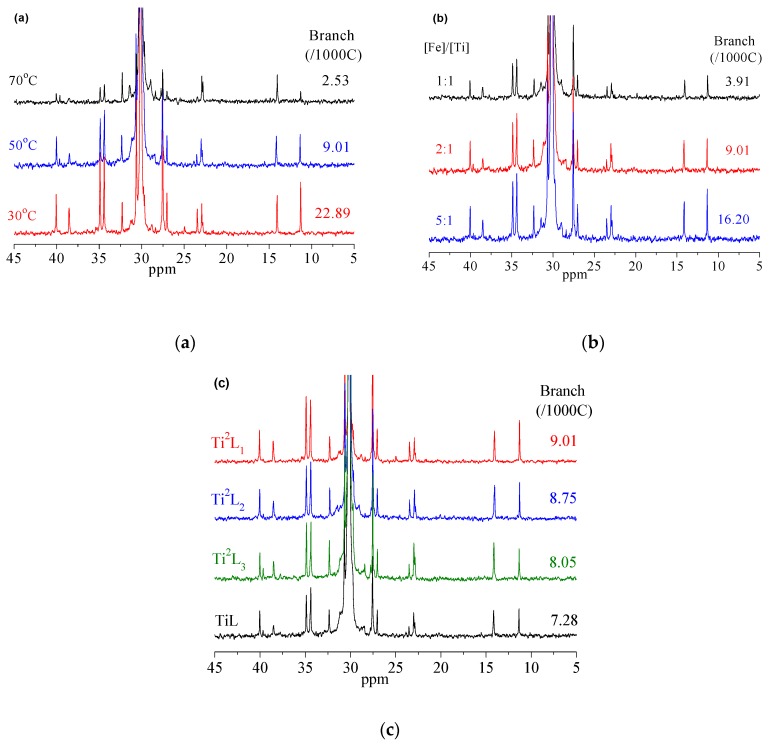
Effects of reaction conditions on ^13^C NMR spectra of LLDPE, (**a**) reaction temperature, (**b**) [Fe]/[Ti] molar ratio, (**c**) different Ti complexes.

The influence of temperature upon the catalytic activities and the structure of polymers were shown in Entries 4–8 of Table 1. The reaction temperature exerted great influence on the catalytic activities as well as the properties of obtained copolymers. When the reaction temperature increased from 30 to 70 °C, the copolymerization activities increased gradually, reached maximum at 60 °C, and then decreased. The trend was in good accordance with the results when catalyzed only by the binuclear Ti complex **Ti^2^L_1_**. However, the branching degree of the obtained copolymers reduced monotonously from 22.89 to 2.53/1000C with the temperature increase from 30 to 70 °C (Figure 3a), presumably due to the decrease of the amount of the α-olefins produced by the bisiminopyridyl iron complex **FeL_a_**. The branches were classified into ethyl, butyl and longer branches according to the ^13^C NMR spectra, and the percentage of different branches among total branches could be separately calculated. The proportion of ethyl branches were more than 50% in all cases, while the percentage of longer branches were generally lower than 20% (Table 2). Short chain 1-butene generally had higher reactivity than the longer chain α-olefins and was much easier to insert into the main chain, moreover, Fe catalyst produced higher proportion of 1-butene at higher temperatures [58], thus higher proportion of ethyl branches were formed.

**Figure 4:**
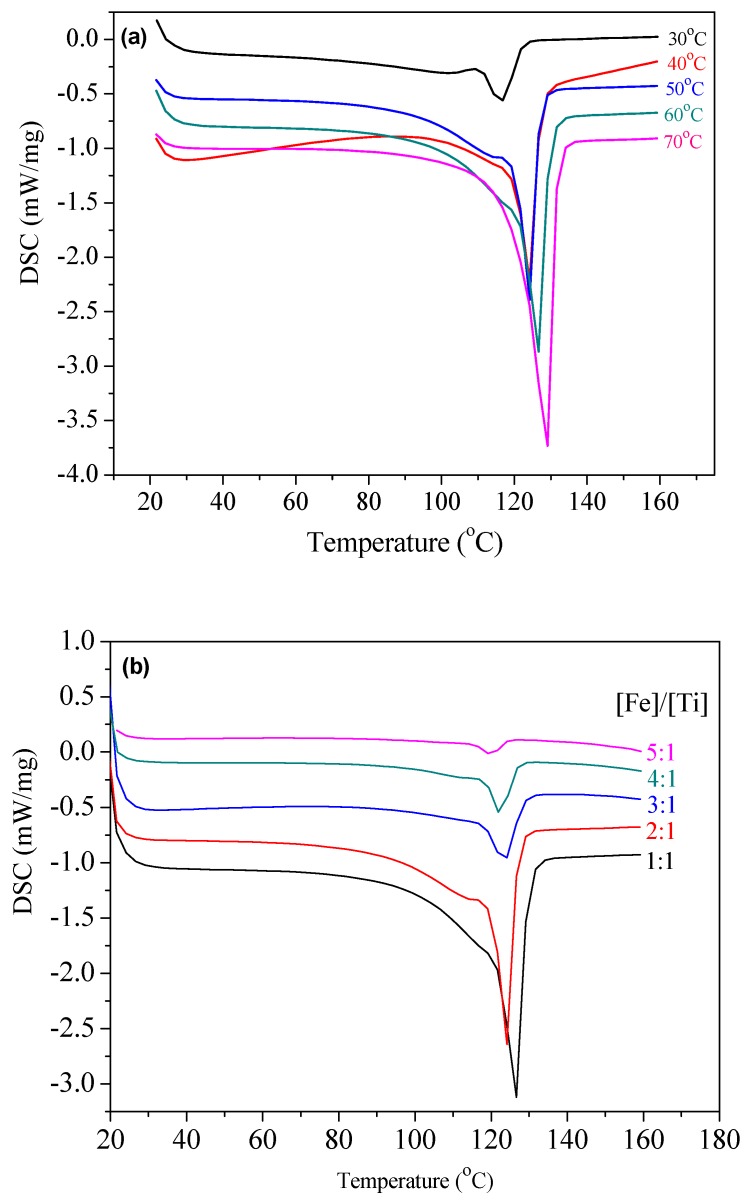
DSC thermograms for LLDPE under different conditions, (**a**) effect of reaction temperature, (**b**) effect of [Fe]/[Ti] molar ratio.

As reaction temperature increased from 30 to 70 °C, the melting temperature *T*_m_ of the obtained copolymers increased from 115.7 to 128.7 °C, and the crystallinity *X*_c_ increased from 2.8 to 53.6% (Figure 4a), further implied that less α-olefins were produced and inserted into the main chain.

**Figure 5:**
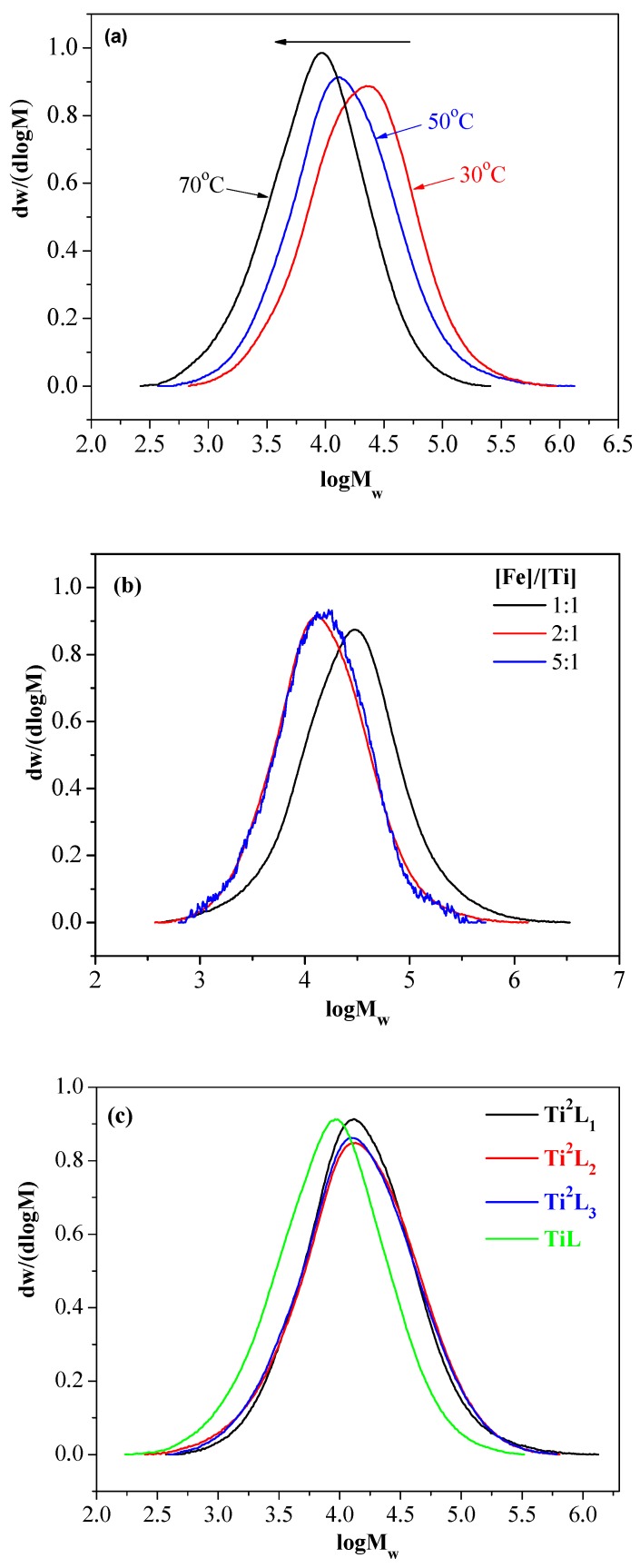
Effects of different conditions on GPC curves for LLDPE, (**a**) reaction temperature, (**b**) Fe/Ti molar ratio, (**c**) different Ti complexes.

The GPC curves of copolymers at different polymerization temperatures were shown in Figure 5a. Increasing the temperatures from 30 to 70 °C decreased the *M*_w_ rapidly from 3.37 × 10^4^ g/mol to 1.30 × 10^4^ g/mol, which was due to faster chain transfer from the titanium species to aluminum and chain termination at higher temperature. The molecular weight distribution remained at a narrow level of 2~3.

### 3.2. Influence of Fe/Ti Molar Ratio

The Fe/Ti molar ratio also influenced appreciably the catalytic behaviors of the tandem catalytic system. Firstly, the catalytic activity increased more than one order of magnitude (0.72 to 8.95 × 10^6^ g/molTi·h·atm) as the amount of Fe catalyst increased fivefold (Fe/Ti molar ratio increased from 1:1 to 5:1). More α-olefins were produced with more Fe complex added, which led to higher calculated activities for Ti complex. These results also fully agreed with the copolymerization behaviors of ethylene and α-olefin catalyzed solely by Ti complex [59]. 

Secondly, it could be reasonably speculated that since more α-olefins was produced with the addition of more Fe catalyst, more comonomers would be incorporated into the polymer backbone, resulting in the increase of branching degree. Indeed, the DSC data in Figure 4b showed that the melting temperature and crystallinity simultaneously decreased from 126.3 to 119.5 °C and from 46.1 to 7.3%, respectively, as Fe/Ti molar ratio increased from 1:1 to 5:1, indicating the continuous increase of branching degrees. The high temperature ^13^C NMR spectra of obtained polymers with different catalyst ratios further confirmed that the branching degrees increased to 16.20/1000C at 5:1 of Fe/Ti ratio, which was almost four times of that at 1:1 Fe/Ti ratio (3.91/1000C), as shown in Figure 3b. The rate of the increase of the branching degree was slightly less than that of the Fe oligomerization catalyst (5 times), but much less than that of copolymerization activity (10 times), which may be a result of the strong positive comonomer effect, i.e., the activity of Ti complex increased faster than the quantity of α-olefins produced by Fe catalyst. Similar phenomenon was also observed in tandem catalysis system consisted of mononuclear nonmetallocene Ti complex and Fe complex [58]. 

Thirdly, the weight-average molecular weights of the copolymers decreased appreciably from 5.41 to 2.34 × 10^4^ g/mol when increasing Fe/Ti ratios from 1:1 to 5:1, due apparently to the incorporation of α-olefins, as shown in Figure 5b.

### 3.3. Influence of the Structure of Ti Complexes

We have also researched the influence of the structures of Ti complexes, including the binuclear Ti complexes bearing different alkylthio sidearms and the mononuclear Ti complex with methylthio sidearm, which were shown in Entries 6 and 12–14 of Table 1 and Table 2.

For binuclear Ti complexes bearing different alkylthio sidearms (Entries 6, 12 and 13 of Table 1), complex **Ti^2^L_1_** with methylthio sidearm exhibited the highest activity and comonomer incorporation ratio, while the Ti complexes with longer alkylthio sidearms (**Ti^2^L_2_** and **Ti^2^L_3_**) exhibited lower ethylene in-situ copolymerization activity and produced polymers with lower branching degrees. The result was fully in agreement with that of ethylene/α-olefins copolymerization catalyzed by the binuclear Ti complexes alone [59], and may similarly be attributed to the steric hindrance of longer alkylthio groups.

The catalytic performances of bi- and mono-nuclear Ti complexes with the same methylthio sidearm were also compared. Both the bi- and mono-nuclear Ti complexes showed excellent performances towards ethylene in-situ copolymerization when combined with Fe complex and activated by MMAO. However, as shown in Figure 3c, the binuclear complex **Ti^2^L_1_** with methylthio sidearm had higher capability for comonomer incorporation and produced copolymers with higher branching degree compared with that by mononuclear analogue (9.01/1000C vs. 7.28/1000C). Furthermore, the molecular weight of copolymers produced by the binuclear Ti complexes was higher than that by the mononuclear complex (2.57 vs. 1.42 × 10^4^g/mol), as shown in Figure 5c. The binuclear Ti complexes had two metal active sites, which would produce two growing carbon chains simultaneously and give rise to larger steric hindrance, reduce the interaction between the complexes and the cocatalyst and inhibit β-Hydrogen elimination and chain transfer reaction; therefore improved comonomer incorporation ratio and increased the molecular weight of copolymers are resulted [59].

## 4. Conclusions

Methylene-bridged tridentate salicylaldiminato binuclear titanium complexes were used in tandem with oligomerization catalyst 2,6-bis(imino)pyridyl Fe complex to produce linear low-density polyethylene (LLDPE) with various branches, including ethyl, butyl and some longer branches by the in-situ copolymerization of ethylene. The binuclear titanium complexes maintained high catalytic activity (over 10^6^ g/molTi·h·atm) and excellent copolymerization capacity when worked together with the Fe complex. The catalytic behaviors and the structures of the obtained polymers were influenced not only by the polymerization conditions such as reaction temperature and Fi/Ti molar ratios but also by the structure of binuclear Ti complexes bearing different alkylthio sidearms. The Ti complexes showed a strong positive comonomer effect and achieved extremely high activity (8.95 × 10^6^ g/molTi·h·atm) with certain Fe/Ti molar ratio. The branching degrees can be adjusted in a wide range from 2.53 to 22.89/1000C by decreasing reaction temperature from 70 to 30 °C, while increasing Fe/Ti molar ratios from 1:1 to 5:1 can increase the branching degrees from 3.91 to 16.20/1000C. The melting points, crystallinity and molecular weights of the products can also be modified accordingly. The binuclear complex **Ti^2^L_1_** with methylthio sidearm exhibited higher capability for comonomer incorporation and produced copolymers with higher branching degree and molecular weight compared with that of mononuclear analogue, as a result of the larger steric hindrance of the binuclear Ti complex.

## Figures and Tables

**Table 1:** Preparation of LLDPE by tandem catalysis of Fe and Ti complexes ^a^.

Entry	Ti Complex	[Fe]/[Ti]	T (°C)	Act.^b^	T_m_^c^ (°C)	*X*_c_^c^ (%)	Branch ^d^ (1000 C)	M_w_^e^	M_w_/M_n_
1 ^f^	**Ti^2^L_1_**	1:0	50	0.78					
2	**Ti^2^L_1_**	0:1	50	0.73	136.4	60.3	0	n.d.	n.d.
3	**Ti^2^L_1_**	1:1	50	0.72	126.3	46.1	3.91	5.41	4.18
4	**Ti^2^L_1_**	2:1	30	0.75	115.7	2.8	22.89	3.37	2.95
5	**Ti^2^L_1_**	2:1	40	0.95	123.9	37.5	n.d.	n.d.	n.d.
6	**Ti^2^L_1_**	2:1	50	1.18	124.2	40.1	9.01	2.57	3.08
7	**Ti^2^L_1_**	2:1	60	1.20	126.4	47.5	n.d.	n.d.	n.d.
8	**Ti^2^L_1_**	2:1	70	0.69	128.7	53.6	2.53	1.30	2.62
9	**Ti^2^L_1_**	3:1	50	1.43	123.3	14.0	n.d.	n.d.	n.d.
10	**Ti^2^L_1_**	4:1	50	3.22	122.3	10.7	n.d.	n.d.	n.d.
11	**Ti^2^L_1_**	5:1	50	8.95	119.5	7.3	16.20	2.34	2.78
12	**Ti^2^L_2_**	2:1	50	1.16	124.4	40.7	8.75	2.54	3.44
13	**Ti^2^L_3_**	2:1	50	0.97	124.7	42.8	8.05	2.50	3.26
14 ^g^	**TiL**	2:1	50	1.84	125.2	44.4	7.28	1.42	3.01

^a^ 40 mL toluene, 10 min reaction time, 0.1 MPa ethylene pressure, 1 µmol Ti complexes, [Al]/[Fe+Ti] = 2000:1. ^b^ Activity, calculated with Ti complex, 10^6^ g/mol(Ti)⋅h⋅atm. ^c^ Measured by DSC, *X*_c_ is degree of crystallinity. ^d^ Calculated by high temperature ^13^C NMR. ^e^ 10^4^ g/mol, determined by GPC using polystyrene standard. ^f^ 4 µmol Fe complex(**FeL_a_**), Activity, 10^6^ g/mol(Fe)⋅h⋅atm. ^g^ 2 µmol.

**Table 2:** Amount of branching in the branched polyethylene.

Entry	Branching with Respect to Total			Branching with Respect to Total [E] Units				Branch/ 1000C
	NE’(%)	NB’(%)	NL’(%)	NE’’(%)	NB’’(%)	NL’’(%)	R (%)	
4	55.45	27.84	16.71	2.54	1.27	0.76	4.58	22.89
6		30.03	12.87	1.03	0.54	0.23	1.80	9.01
8	80.12	15.66	4.22	0.41	0.08	0.02	0.51	2.53
3	72.15	21.94	5.91	0.56	0.17	0.05	0.78	3.91
11	57.87	30.96	11.17	1.87	1.00	0.36	3.24	16.20
12	51.61	26.73	21.66	0.90	0.47	0.38	1.75	8.75
13	62.35	19.14	18.52	1.00	0.31	0.30	1.61	8.05
14	62.38	29.47	8.15	0.91	0.43	0.12	1.46	7.28
